# Standardizing the Development of Serious Games for Physical Rehabilitation: Conceptual Framework Proposal

**DOI:** 10.2196/25854

**Published:** 2021-06-24

**Authors:** María Del Pilar Beristain-Colorado, Jorge Fernando Ambros-Antemate, Marciano Vargas-Treviño, Jaime Gutiérrez-Gutiérrez, Adriana Moreno-Rodriguez, Pedro Antonio Hernández-Cruz, Itandehui Belem Gallegos-Velasco, Rafael Torres-Rosas

**Affiliations:** 1 Doctorado en Biociencias Facultad de Medicina y Cirugía Universidad Autónoma “Benito Juárez” de Oaxaca Oaxaca de Juárez Mexico; 2 Escuela de Sistemas Biológicos e Innovación Tecnológica Universidad Autónoma “Benito Juárez” de Oaxaca Oaxaca de Juárez Mexico; 3 Facultad de Ciencias Químicas Universidad Autónoma “Benito Juárez” de Oaxaca Oaxaca de Juárez Mexico; 4 Laboratorio de genómica y proteómica Centro de Investigación UNAM-UABJO Facultad de Medicina y Cirugía UABJO Oaxaca de Juárez Mexico; 5 Laboratorio de Inmunología Centro de Estudios en Ciencias de la Salud y la Enfermedad, Facultad de Odontología Universidad Autónoma “Benito Juárez” de Oaxaca Oaxaca de Juárez Mexico

**Keywords:** serious game, physical rehabilitation, framework, software engineering, gamification

## Abstract

**Background:**

Serious games have been used as supportive therapy for traditional rehabilitation. However, most are designed without a systematic process to guide their development from the phases of requirement identification, planning, design, construction, and evaluation, which reflect the lack of adaptation of rehabilitation requirements and thus the patient’s needs.

**Objective:**

The aim of this study was to propose a conceptual framework with standardized elements for the development of information systems by using a flexible and an adaptable process centered on the patient’s needs and focused on the creation of serious games for physical rehabilitation.

**Methods:**

The conceptual framework is based on 3 fundamental concepts: (1) user-centered design, which is an iterative design process focused on users and their needs at each phase of the process, (2) generic structural activities of software engineering, which guides the independent development process regardless of the complexity or size of the problem, and (3) gamification elements, which allow the transformation of obstacles into positive and fun reinforcements, thereby encouraging patients in their rehabilitation process.

**Results:**

We propose a conceptual framework to guide the development of serious games through a systematic process by using an iterative and incremental process applying the phases of context identification, user requirements, planning, design, construction of the interaction devices and video game, and evaluation.

**Conclusions:**

This proposed framework will provide developers of serious games a systematic process with standardized elements for the development of flexible and adaptable software with a high level of patient commitment, which will effectively contribute to their rehabilitation process.

## Introduction

### Background

Human motor skills can be affected by numerous adverse situations such as trauma, stroke, and degenerative diseases. Rehabilitation exercises play a fundamental role in reducing the degree of disability. The traditional assisted rehabilitation model consists of daily supervised exercise sessions with a therapist [1]. These exercises must maintain patient motivation through interactive and stimulating environments to be effective. The therapist must customize rehabilitation exercises according to the patient’s needs. Many rehabilitation therapies are intense and involve numerous repetitions and exercises. Patients often experience frustration owing to mobility loss. It leads to states of depression, causing some patients to become discouraged and lose interest in therapeutic exercises [2-4]. To avoid this, some complementary techniques combined with traditional rehabilitation, such as serious games, allow enhanced recovery [5]. They offer a more attractive environment and maintain the interest in the process of motor rehabilitation, focusing on the game instead of its limitation [6]. Serious games are video games that are meant for education instead of entertainment [7]. Therefore, the game must impart additional experiences to the user in addition to knowledge or skills. Although several serious games for rehabilitation have been developed [5,8-10], there are still misunderstandings in spite of the systematic and standardized process for their creation. These misunderstandings cause the inadequate implementation of important elements such as specialist monitorization, motivation, game levels, and evaluation scales to objectively quantify the degree of the disability. This paper explains the creation of a conceptual framework with a systematic, standardized, flexible, and adaptable approach for the development of serious games in physical rehabilitation. A conceptual framework helps synthesize the knowledge of different areas to obtain a broad understanding of the topics [11]. This framework is based on the structural activities of software engineering applied in a user-centered design (UCD) approach with an iterative and incremental process that allows the visualization of prototypes from the beginning of development with gamification elements to increase commitment and motivation.

### Related Works

Previous studies have developed serious game frameworks in various areas such as physical rehabilitation and education. The following papers were obtained when reviewing the existing literature.

Amengual et al [12] proposed a system based on a two-dimension (activities and incremental development) iterative process. It consists of 4 phases: project initiation, interaction elements, serious games, and evaluation. In project initiation, therapists identify the patients’ needs. The interaction mechanism for the patient’s movement is then selected. A serious game is created, and finally, the patient is clinically evaluated. The framework is based on (1) scrum to manage and control iterative work at a project level, (2) a web application development model because the authors consider its requirements to have a certain similarity with web development, and (3) a process that requires a set of sequential phases, where each phase attempts to meet or define some objective because as per Amengual et al [12], the development of a serious game for motor rehabilitation is similar to that of a clinical trial.

Ushaw et al [13] proposed a paradigm identifying a benefit delivery system for serious games. It is classified into 5 elements: repetition, exploration, strategy, progress, and social interaction. They proposed a triangle for resources (time), benefit (serious), and game (fun) based on the “iron triangle” of software development, which is focused on quality. The application development phase is carried out with design, implementation, testing, and assessment phases.

Olszewski et al [14] proposed a structured framework for game development in medical education. It is an iterative process comprising 3 phases of development (preparation, design, and development) and a formative evaluation process. In the preparation and design, a team of medical experts is created according to the serious game developed. They will state the necessary knowledge to the development team. A design script is created, visualizing the hospital room and the game organization through navigation elements. In the development phase, the illustration components of the game are made to improve visual communication interface learning. Prototypes are created for a team of experts to analyze and make adjustments. During evaluation, formative, design problems, functionality, and usability problems are identified in the game. Then, the finalized project is delivered.

Pirovano et al [15] proposed a four-step framework. The first step is exercise, which begins with the therapy goal through exercises and is classified as primary and secondary goals. The second step is virtualization. The primary goals are turned into virtual exercises. In the third step, the virtual exercise becomes a real serious game. Finally, the secondary goals are managed through a monitoring module to adjust the patient’s progress.

Table 1 summarizes the characteristics of each framework with structural activities and identifies the gamification elements. None of the studies in the literature review proposed activities to build an interaction device.

The main differences between our framework and that mentioned in similar studies are as follows: (1) our framework contains 5 structural activities of software engineering applied to a UCD; (2) physical rehabilitation–oriented gamification elements were included and classified into 3 groups (flow enhancement, immersion, and progress), which are implemented in the design phase to motivate the patient and to generate an immersive environment, thereby preventing dropouts; and (3) we propose a phase to develop a data acquisition interface to process the patient’s movements when commercial devices do not adapt to the rehabilitation process.

**Table 1:** Summary of the related studies.

Framework	Structural activity	Gamification elements	Information on interaction device
Amengual et al [12]	Project initiation Planning and control (communication and planning) Modeling Construction Evaluation (deploy)	Levels	No
Ushaw et al [13]	Serious goal and game-play mechanic (communication and planning) Design (modeling) Implementation (construction) Testing assessment (deploy)	Benefit delivery mechanic: repetition, exploration, strategy, reward, measurement	No
Olszewski et al [14]	Preparation (communication) Design (modeling) Development (construction) Formative evaluation (deploy)	—^a^	No
Pirovano et al [15]	Exercise definition (communication) Virtualization (modeling) Primary and secondary goals (modeling and construction) Game design (construction and deploy)	Feedback and motivational factors	No

^a^Not available.

## Methods

### Study Design

**Figure 1:**
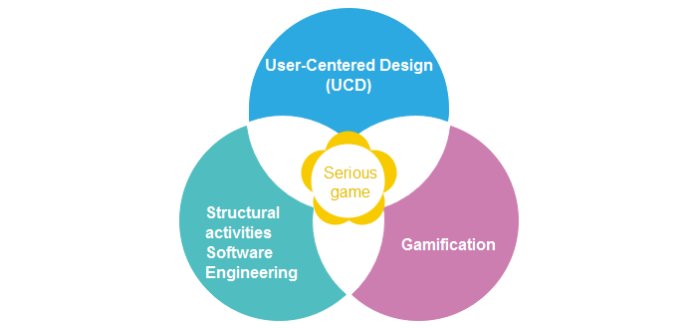
Conceptual elements of the framework.

Our conceptual framework is based on 3 fundamental concepts: (1) software engineering because serious games are based on the principles of information systems; (2) UCD, which is an iterative design process that focuses on users and their needs in each phase of the project; and (3) gamification, which allows the transformation of obstacles into positive and fun reinforcements, thereby encouraging patients in their rehabilitation process. The concepts used in our framework proposal are shown in Figure 1.

### Framework in Software Engineering

The software engineering framework establishes a high level of abstraction for software development, applying concepts, models, and other elements. It provides solutions to a series of similar problems, generally describing the phases that must be followed to fix them without further detail of the activities in each phase [16]. The objective is that developers use the framework as a guide for the creation of software systems, applying its phases as “building blocks” depending on the problem.

### Structural Activities in Software Engineering

The work associated with the development of information systems in software engineering is classified into generic structural activities [17,18], regardless of the field of application, project size, or complexity. The structural activities are communication, planning, modeling, construction, and deployments, which are defined below:

1. Communication: This activity focuses on identifying the context and key requirements of the system through collaboration between the client and the development team. This phase determines the information processed, developed interfaces, design restrictions, and validation criteria.
2. Planning: This activity identifies requirements and develops resource estimates. Development tasks are identified and a work plan is created. Then, techniques are applied to define a work path and the strategic goal of the project.
3. Modeling: With a multidisciplinary team, the models must understand the real entity and represent the characteristics that the users need in addition to the information obtained and transformed with the software. The models must meet these objectives at different abstraction levels, including the illustration of software from the user perspective and on a technical level for the development team.
4. Construction: In this activity, models are coded in a programming language, errors are detected through tests, and they are corrected, resulting in a smart operating software for the client or end user.
5. Deployment: The prototype is delivered to the end user. The customer must provide feedback on the project for improvements. The software development process is iterative and incremental, and as a result, several deployments are made until the software development is completed.

These 5 generic structural activities are used during software development. The process details will be different in each case, but the structural activities remain the same.

### UCD

UCD is an iterative design process that focuses on users and their needs in each phase of the design process. Users are involved during the design process through research and design techniques to create highly usable and accessible products for them [19]. According to Karat and Karat [20], “UCD is characterized as a multi-phase problem-solving process that requires designers, analysis and foreseeing of the product or service employment, and verifying the validity of the behavioral assumptions in the real world.”

**Figure 2:**
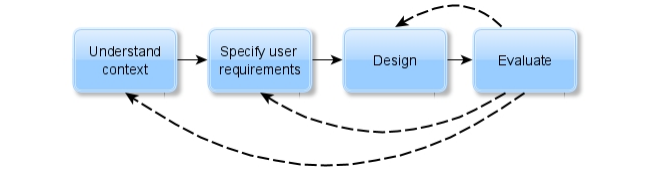
User-centered design process.

UCD is an iterative process that includes the following key principles [21]: end users must be actively involved from the onset, and throughout the life cycle, the development comprises many iterative and incremental cycles to meet the end user requirements; design and prototypes must be created early and continuously to help visualize and evaluate ideas; and the development process must be performed by interdisciplinary teams. The principles described above facilitate the development, communication, and evaluation of the UCD to create interactive and useful systems, covering the design, evaluation, construction, and implementation phases of the product. Each UCD iteration involves 4 phases: contextual analysis, the definition of requirements, design, and evaluation. In the contextual analysis, the designer team must understand the context in which the system is used. When defining requirements, these are identified and specified according to the user. In the design phase, the team develops solutions such as simplified prototypes and designs on paper. In the last phase, results are evaluated from the assessment of the context and user requirements, verifying the design performance and satisfaction of relevant user needs. Depending on the results, the project team takes up phases again to optimize the product. These repetitions are performed until a satisfactory response is obtained from the users. Figure 2 shows the UCD process.

### Need of a UCD for People With Disabilities

Helander and Landauer [22] established that people with disabilities have similarities with older adults since “they have multiple physical problems with a general reduction of their functionalities.” People with special needs such as mobility limitations require an adaptable rehabilitation process for their needs. Thimbleby [23] stated that the purpose of UCD for a user with special needs is to increase their work productivity. Thus, the design must have end-user acceptance as they can feel more comfortable using the end product. To perform a physical rehabilitation process through serious games, the patient’s movement to control the video game should be obtained. Therefore, the interaction device must be easy to wear and match the motor capacity of the patient. For example, if the therapy requires finger movement, it is difficult to put a haptic glove on the user (which could be a nuisance). Thus, the use of optical devices such as cameras and computer vision techniques are preferred.

### Gamification

Gamification is a relatively new concept. Its objective is to apply game mechanics in different contexts to attract users to mundane but fun activities with motivational and cognitive benefits [24]. It allows the transformation of obstacles to positive and fun reinforcements, thereby encouraging users in making the right decisions for their health and well-being [25]. Several authors have proposed gamification elements for serious games, which are described below.

Cheek et al [26] identified design elements in a serious game called SPARX for adolescents with depression with a user-centered perspective. They identified 4 important areas and a series of associated elements: computer games (challenge, companionship, exploration, fantasy, and fidelity), accessibility (perceivable information, operable interface, and understandable, robust, and reliable information), working alliance (goal, task, and bond), and learning in immersion (situational learning, multiple perspective, real-life simulation, and immersive factors).

Zain et al [27] introduced a framework based on the flow theory of computer game usability and user experience. This framework consists of 8 elements: player skills, challenge, concentration, feedback, immersion, learning opportunities, accessibility, and adaptability.

Specifically, Schulz et al [28] proposed a series of design element specifications based on functional and professional requirements: immersion, support for different roles, flow enhancement, visual enhancement, support for different learning phases and experience levels, design for interactivity, and progress.

Vermeir et al [29] presented a systemic review and meta-analysis of the gamification effects on computerized cognitive training. The elements identified were an avatar, challenge, competition, difficulty adjustment, feedback loops, levels, progress, rewards, social interaction, sound effects, and story/theme.

Bergeron [30] proposed a series of design elements: concept, game features, setting, story and backstory, effectors, game flow, screens and menus, control, options, sound and music, levels, score tracking, help, and localization.

**Figure 3:**
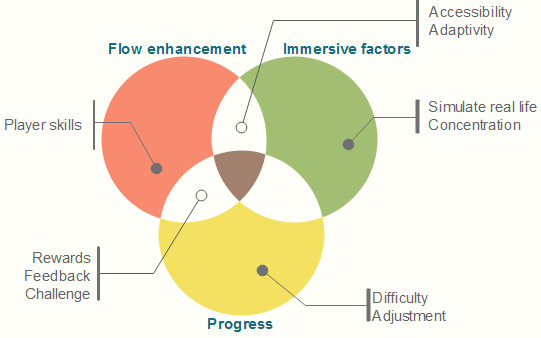
Classification of gamification elements.

From the gamification elements mentioned before, we chose those shared in every study and those that were convenient in a serious game for physical rehabilitation, and we classified the elements into 3 groups: flow enhancement, immersive factors, and progress. These concepts may appear in more than one group. For example, the element “rewards” is included in flow enhancement and progress. The shared gamification elements are shown in Figure 3.

1. Challenge: According to Zain et al [27], the game must be challenging enough, and it has to match the player’s skill level. In physical rehabilitation, the game must adapt to the patient’s possibilities and be challenging enough to prevent boredom.
2. Accessibility: This element refers to the capacity to adapt to the patient’s disabilities. For example, when identifying hand movements in a patient who cannot hold an object, a camera can be used to track movements or a device that can be held by the patient.
3. Adaptability: According to Zain et al [27], the user needs 3 factors: (1) user motivation, that is, why are you interested? (2) experiences and skills, that is, what skills are required to play? and (3) detection, that is, identify when a level change is necessary.
4. Player skill: The skill must be consistent with the serious game. As the game progresses, the patient develops more skills that motivate him/her to continue the rehabilitation process.
5. Rewards: Use indicators of the patient’s progress such as points, virtual coins, badges, or any virtual object to motivate the user to continue with the rehabilitation process.
6. Real-life simulation: Games simulating real-life activities allow patients with physical disabilities to immerse themselves in the game.
7. Concentration: A serious game is motivating when the patients can fully focus on the game. Zain et al [27] indicate that serious games should attract the patient’s attention at all times, avoiding distractions from the main task.
8. Feedback: There are different ways to provide feedback to the patient: (1) through progress, when the patient has correctly performed the exercise and must be motivated; (2) when indicating how to correctly perform an exercise; (3) through rewards with badges or virtual gifts when completing a challenge.
9. Difficulty adjustment: The serious game must be developed such that it allows the therapist to indicate the start level and make the necessary adjustments to the rehabilitation exercises.

## Results

### Proposal of a Conceptual Framework

**Figure 4:**
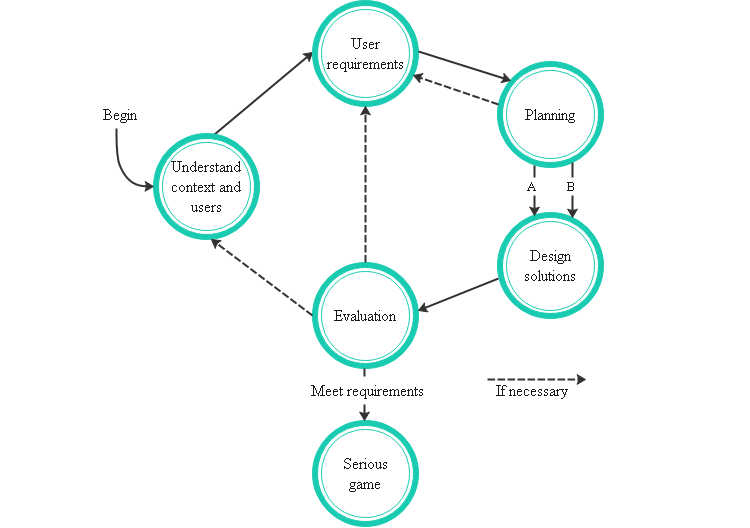
Proposed conceptual framework for the development of serious games for physical rehabilitation.

Few studies use a framework to develop serious games systematically. Therefore, our objective was to propose a conceptual framework based on UCD. Our framework consists of the adequate application of gamification elements and structural activities and guidance of meaningful, pleasant, relevant, and motivating serious games for physical rehabilitation. We use certain phases of the original UCD process, including a planning phase to establish estimates and priorities of the requirements and a modified designing phase to identify between creating an interaction device or using a commercial one. Figure 4 shows and describes the conceptual framework and the relation between the phases.

**Figure 5:**
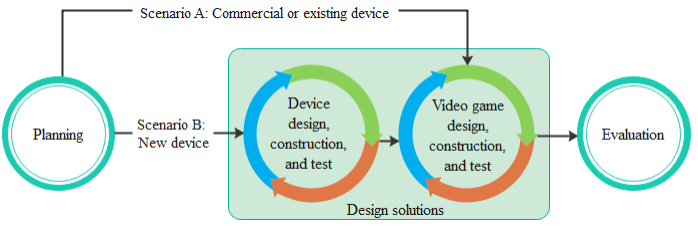
Contemplated scenarios.

The framework begins in the phase of context and user understanding, and then the user requirements are identified. When new requirements are needed in the planning stage, it is necessary to return to the previous phase and include them in the user requirements. From planning to designing solutions, there are 2 possible scenarios shown in Figure 5: scenario A is applied when an existing or a commercial device is being used and scenario B is applied when creating a new device for a serious game. The evaluation has 3 options: (1) user requirements, in case of changes or improvements in the prototype; (2) context and user understanding, when clarifying the context for a requirement that needs to be adapted; and (3) serious game development when requirements are met, and the project satisfies the users’ needs.

### Define Use of Context and Users

When developing a system or product, certain characteristics must be considered, such as context and user population with specific goals and tasks. Other conditions are technical, physical, social, or organizational that may affect its use. The quality of use of a system, user-friendliness, user health, and safety will depend on having an adequate understanding of the context. Identifying the correct context will help specify the user requirements and provide a solid foundation for subsequent evaluation activities. For well-known systems, the identification of stakeholders and context use review is sufficient. Further analysis of context and a study of existing users is required for more complex systems.

#### User Identification

The identification of direct and indirect users (people who influence or are affected by the system) ensures that every need is met and is tested as its construction progresses. User Mapping is a tool to identify users, as proposed by Taylor et al [31].

#### Analysis of the Context of Use

There are structured methods that obtain detailed information to understand the context of system use as a foundation for subsequent usability activities, particularly the specification and evaluation of user requirements. Some methods for context analysis have been proposed by Maguire [32], Taylor et al [31], and Thomas and Bevan [33]. For example, a guidebook for context analysis was developed by Thomas and Bevan [33], while Taylor et al [31] described the background and importance of understanding the context of use by developing a set of tools to identify the types of users, their needs, characteristics, and translation of this information into user requirements. This method is especially directed to nonexperts in the area of UCD and evaluation.

### User Requirements

This stage identifies and documents the potential user requirements derived from the context information. Establishing and documenting user requirements will lead to the design process of a system [17]. User requirements include summarized descriptions of the system tasks and the features provided to support them. Therefore, user requirements describe the system characteristics to meet the context of use characteristics. Requirements engineering is needed to carry out this phase. It establishes a process of discovering, analyzing, documenting, and verifying requirements. Requirements engineering can be described in 5 distinct steps: requirement elicitation, requirement analysis and negotiation, requirement specification, system modeling, requirement validation, and requirement management [17]. Other techniques are proposed by Saiedan and Dale [34]. Once the requirements are obtained, they are analyzed with everyone involved. Then they must be documented with a user requirement(s) document or software requirements specification. An example of documenting requirements is the user stories used in the agile methodology XP [35].

An essential requirement in physical rehabilitation is checking the patient progress and matching their levels. For example, the Wolf motor function test [36] or Fugl Meyer assessment [37] is used for upper limbs, the Berg Balance Scale [38] for balance and posture, and the Lower Extremity Motor Coordination Test for lower limbs [39]. These scales can be applied by the therapist or can be automated in the serious game. The latter must be added as a specific requirement for patient evaluation through the activities. The developed requirements are selected in each iteration. The requirements are adaptable to changes with the possibility of adding or removing them at any stage according to the system’s needs. The iterations will conclude when the user requirements end. Measurement and compliance of the user requirements during development will result in a successful serious game that will improve patient safety, treatment effectiveness, and reduced rehabilitation time.

### Planning

Several authors [17,18,40-42] have mentioned that planning is an essential part of project development. The work must be divided and assigned to the team members. Planning allows goal definition, objectives, and path to follow. The project size must be independently established with a project plan [43] containing at least the following elements [17,43]: team organization, risk analysis, requirements and estimation of resources, work division, and project schedule.

**Figure 6:**
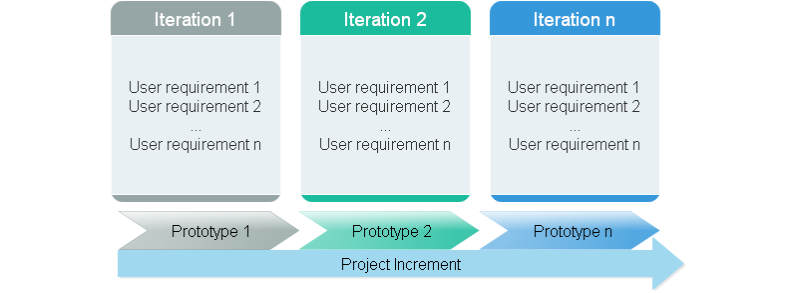
Initial planning of user requirements.

Once the user requirements are established, they must be divided by iterations to obtain a prototype in each cycle, as shown in Figure 6. Pressman [18] indicated that planning should be iterative and repeated at the end of each iteration based on therapist and patient feedbacks. Thus, planning is repeated in each iteration as user requirements are defined in the iteration, and time and resources are identified to successfully conclude the prototype. Planning should also be frequently monitored, and adjustments made as required. Assessing the progress daily will detect problem situations and adjust the plan accordingly.

### Design Solutions

Designing is a creative activity where components and their relationships are identified based on user requirements. The team approaches designing through different solutions, and every idea must go through iterative development. The product meets the potential user needs through its development with some design elements such as mock-ups or interface screens for interaction, visualization, or comments. Another formal modeling such as UML [44] must be used by developers to represent the parts and communication of the system. Design changes can be made quickly in response to user feedback, and significant design issues can be identified before the system development begins. The solution is subsequently proposed through the prototypes. Hall [45] states the development of at least a low fidelity (for example, mock-ups) and a high fidelity (operational system, simulation) prototype. This will allow a usable product to satisfy the user requirements. Finally, tests must be run and possible errors must be corrected.

### Device Design, Construction, and Test

Two possible scenarios are established in this phase, which are described below:

#### Scenario A: Use of an Existing or a Commercial Device

**Figure 7:**
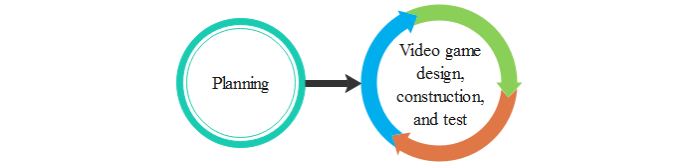
Scenario A: Use of an existing or commercial device.

Devices previously created from an iteration or commercial devices such as Microsoft Kinect, Leap Motion, and Novint Falcon Game Controller are used in many serious game developments. The development team must ensure that it is safe and meets the patient’s needs. Once the interaction device is selected, the creation phase of the video game is initiated. Figure 7 shows the transition phase.

#### Scenario B: New Device Development

**Figure 8:**
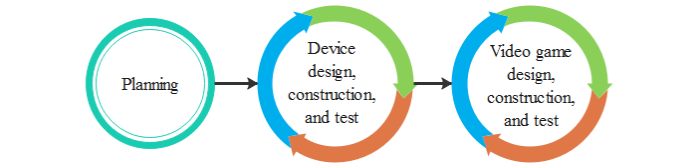
Scenario B: New device development.

This scenario occurs when the commercial device does not allow complete customization, and there are limitations in the data processing. This requires an additional phase to create personalized devices (eg, exoskeletons, gloves with inertial sensors) that match the motor skills of the patient for game movement and control. Figure 8 shows the phase to create a new device. Design in this phase creates models for the development team to understand the new device requirements. The models (for example, wireframes or mock-ups) help obtain comments and feedback for a better understanding of the operation. Devices are produced as a result of adequate designs in the construction phase [45]. The most appropriate components must be selected from the needs and limitations of the patients. Once the components are assembled, data processing is performed, including input, process, and output. Finally, the test activity allows testing and correcting possible errors to determine if the device meets the requirements.

### Design, Construction, and Testing of the Video Game

#### Design of the Video Game

In this phase, the video game prototype is developed, which is controlled by the interaction device of the previous phase. The previously analyzed and planned requirements are used in this phase. Regardless of the software’s scope, size, or complexity, the software design must include at least four of the following models: data or class design, architecture design, interface design, and component-level design [18]. In data design, the data structures that will be required to implement the software are created, and data objects and relationships are defined. The architectural design defines the relationship between the major structural elements, and the “design patterns” can be used to achieve the requirements that have been defined for the system. The interface design describes the flow of information and how the software communicates within itself, with other systems that interoperate with it, and with humans who use it. The component-level design transforms structural elements of the software architecture into software components. Furthermore, game design elements are important to be included, which are described below.

##### Game Design Elements

In the Methods section, gamification was described and classified into 3 aspects: flow enhancement, immersive factors, and progress. Table 2 shows the benefits of gamification from game design elements. Therefore, it is of importance to include the following design elements:

###### Game Genre

Different game genres include action, adventure, music, puzzle, role-playing game, simulation, and strategy. The genre must be appropriate to the age of the patient in rehabilitation. For example, Chesham et al [46] indicated that puzzles are easy to understand, learn, and play for older adults.

###### Story or Narrative

According to Kuiper [47], a story is a series of events organized in a temporary order. According to Lu et al [48], the narrative influences the patient’s cognition, affection, and potentially healthy behavior of the players. The story must be written according to the patient’s average age and body part in rehabilitation.

###### Actors

According to Bergeron [30], primary actors in most games are player character(s), nonplayer character(s), vehicles, and effectors. Defining actors according to the rehabilitation process, history, and average age of the patient is important.

###### Effectors

Effectors are instruments that players have to interact with other game elements or to complete a mission. They are closely related with the interaction device (device that follows the patient’s movements) and used in the rehabilitation process. They must agree with the story and avatar.

###### Screen and Menus

Bergeron [30] stated that an action or role-playing game must include startup, main menu, inventory, level, exit, and high-score screens. Screens and menus must be adapted to the physical limitations of the patient, for example, fine motor skill problems preventing the use of a mouse.

###### Levels

Baranowski et al [49] indicated that levels help players view their progress, thereby allowing the dominance of an action before moving to the next level. Levels must be associated with patient recovery in physical rehabilitation. Evaluation scales must be included to measure the patient’s progress in a standardized way.

###### Help

Help shows game instructions to the user. It can be a small guide describing the movements to control the game or a document with frequently asked questions.

###### Sound and Music

Define different sounds according to the game’s context. Bergeron [30] stated that music must be according to a particular situation, for example, when transmitting emotions such as happiness. Typical musical cues are needed for the introduction, level ending, high score, victory, and defeat.

###### Visual Enhancement

According to Schulz et al [28], games use visual cues to guide a player and provide options or interactive elements. Visualization helps patients become familiar with an environment, identify a real-life–like scenario, and intuitively select the effectors needed to accomplish a task. For example, if a player is presented with a dirty window on the screen, they will take a glass cleaner to clean it.

**Table 2:** Benefits of gamification through game design.

Game design	Gamification benefits
Game genre	Flow enhancement, immersive factors
Story or narrative	Flow enhancement, immersive factors, progress (challenge)
Actors	Flow enhancement, immersive factors (simulate real life)
Effectors	Immersion (simulate real life, accessibility), flow enhancement
Screens and menus	Flow enhancement (challenge, accessibility, rewards), progress
Levels	Flow enhancement, progress
Help	Flow enhancement (player skills), immersive factors (accessibility)
Sound and music	Immersive factors (adaptivity), feedback, rewards
Visual enhancement	Immersive factors (simulate real life, concentration), player skills, rewards, feedback

#### Construction of the Video Game

Software components, data, library, and other items are assembled at this stage to compile and link them to create an executable system.

#### Testing of the Video Game

Testing units may discover program defects before use. It has 2 distinct goals [17]: (1) show that the software meets the requirements of the development team and client and (2) find situations of wrong software behavior or not according to the specifications.

### Evaluation

User-controlled testing is the most adequate method of assessment [50-52]. It consists of configuring system tests to perform a series of tasks by representative users. This can be configured in a controlled laboratory environment or with the developers. The objective is to collect information from the user’s performance with the system, feedback, reactions, and observations. Another method is satisfaction questionnaires [53,54] with subjective impressions based on experiences with the system or a new prototype. Controlled clinical studies are recommended in the evaluation phase [55] to quantify the rehabilitation improvement with the exercises. The experiment, participants, and measurements must be defined according to the type of therapy [56]. If patients or therapists detect problems in the prototype created in an iteration, it must be solved in the previous iteration of the requirement or user context phase.

## Discussion

### Main Findings

The development of serious games for physical rehabilitation is a multidisciplinary process involving several elements: software development, design aspects, and direct involvement of health care specialists, patients, and other nonprofessional health care personnel. Although multiple developments have used UCD [57-60], they do not apply the structural activities required for a software system development [17,18,41,42]. Gamification allows obstacles to transform into positive and fun reinforcements in a physical rehabilitation process. The proposed framework considers gamification strategies and ensures their fulfillment with game design elements. This strategy is innovative since a similar proposal is not found in related literature. The reviewed studies described the concept but not the application of gamification in a development phase [26-28]. Serious games require a communication interface to control the video game. In physical rehabilitation, following a particular movement in a patient’s limb or specific injury is required. Therefore, this conceptual framework includes scenarios to select the most appropriate device, including a commercial or existing device or the creation of a custom device. The frameworks of the reviewed studies did not consider the use or creation of interaction devices, as shown in Table 1.

### Limitations

The authors acknowledge the limitations of this conceptual framework, such as validation, which has to be applied to patients requiring physical rehabilitation, and the generality in its description. However, the latter has the objective to provide a generic framework for physical rehabilitation with an understandable approach to development teams of serious games.

### Opportunities for Further Research

This conceptual framework will be implemented in a serious game prototype application involving a health expert throughout the development process and validated by statistical analysis and clinical evaluation of patients.

### Conclusions

Most serious games do not use a systematic process for their creation, thereby producing significant omissions in the rehabilitation process such as lack of rating scales to measure the patient’s progress, no feedback, and exercises that do not adapt to the patient’s disabilities. Therefore, this study provides a systematic process for the development of serious games for physical rehabilitation with the proposal of a conceptual framework. The framework applies 3 key concepts that increase the patient’s adherence to rehabilitation therapy: UCD to understand the specific needs of patients, structural activities of software engineering for their development, and gamification elements, which aim to influence the behavior and motivation of users through the experiences obtained in the game. Access to this type of framework will assist development teams in the creation of safer, fun, motivating serious games, thereby improving the participation and commitment of patients. Finally, it would be essential that every serious game published in a journal be developed through a standardized process applying a framework, thereby ensuring that the game meets the minimum requirements necessary to satisfy user needs.

## Abbreviations

UCD: user-centered design
